# CD146 T cells in lung cancer: its function, detection, and clinical implications as a biomarker and therapeutic target

**DOI:** 10.1186/s12935-019-0969-9

**Published:** 2019-09-26

**Authors:** Ayobami Matthew Olajuyin, Adefunke Kafayat Olajuyin, Ziqi Wang, Xingru Zhao, Xiaoju Zhang

**Affiliations:** Department of Respiratory and Critical Care Medicine, Henan Provincial People’s Hospital, People’s Hospital of Zhengzhou University, Zhengzhou, 450003 Henan China

**Keywords:** CD146, T lymphocytes, Lung cancer, Biomarker, Therapeutic, *Calotropis procera* leaf

## Abstract

CD146 alternatively called melanoma cell adhesion molecule (MCAM), is a biomarker and therapeutic target of clinical significance. It is found on different cells including the endothelial cells and lymphocytes which participate in heterotypic and homotypic ligand-receptor. This review concentrated on the CD146 expression T cells (or lymphocytes) centering on Treg in lung cancer. Here, we have also considered the vigorous investigation of CD146 mainly acknowledged new roles, essential mechanisms and clinical implications of CD146 in cancer. CD146 has progressively become a significant molecule, particularly recognized as a novel biomarker, prognosis and therapy for cancer. Hence, targeting CD146 expression by utilization of methanol extracts of *Calotropis procera* leaf may be useful for the treatment of carcinogenesis.

## Background

CD146 is a cell adhesion molecule (CAM) which was first discovered in 1987. It is 113,000-daltons membrane glycoprotein that comprises of transmembrane region, five immunoglobulin-like domains and a short cytoplasmic tail [1]. CAMs are utilized in a wide array of pathophysiological processes such as apoptosis, cell cycle, cell migration, cell–cell and cell–matrix interactions, cell signaling and morphogenesis during growth and tissue remodeling. Cell adhesion is an important process necessary for the accurate performance of eukaryotes. Researchers have shown the roles of CAMs in diversity of pathological progressions in cancer, pulmonary hypertension, autoimmune diseases, inflammation and infections [2, 3]. CD146 is known to be a member of the CAM because of its sequence homology analysis. It is a well-known adhesion marker of endothelial cells [4], which has also been recognized on some other cell types such as lymphocytes, pericytes, immune cells, mesenchymal stem cells, human alveolar periosteal sheets, bone marrow fibroblasts etc. [5–9]. It has been studied extensively in circulating endothelial cells [10, 11]. Hence it is called MCAM (melanoma cell adhesion molecule). Emerging researches have shown CD146 is expressed on different types of lung cancer [12–15]. Therefore, CD146 may be a possible biomarker for tumor diagnosis, therapy and prognosis.

Lung cancer remains the noticeable reason for cancer mortality globally [16] and the second most widespread cancer in *homo sapiens* [17]. Medically, the diagnosis of lung cancer is very dismal. Nevertheless, most cases of the advanced-stage lung cancers are very common, and investigations are going on seriously. However, the prognosis for patients with lung cancer remains unfavorable [18]. Hematologic irregularities, including anemia, thrombocytosis leukocytosis and lymphopenia are frequently observed in lung cancer patients. In the healthy subjects, the expression of CD146+ T cells is between 1 and 3% in the blood. However, the expression in a disease state such as lung cancer is significantly increased compared to the healthy patient [6]. CD146+ T cells have improved the interaction to endothelial monolayers, have effector memory phenotype, T regulatory phenotype, in adhesion, several genes are involved such as galectin 1 (LGAL 1), galectin 3 (LGAL 3), translocation, and inflammation, which may protect apoptosis [19]. These characteristics of the CD146+ T cells in the peripheral blood have steered to the assumption that these may demonstrate a minor pool of cells for homing of activated T cells [19, 20] in retort to inciting stimuli. The expression of CD146+ T cells in lung cancer and autoimmune diseases patients are said to be elevated [21–23]. The significance of CD146 T cells at the site of inflammation in these diseases remains unexplored.

*Calotropis procera* (CP) is a xerophytic perennial shrub which is found majorly in subtropical and tropical Middle East, Asia and Africa [24]. Various parts of CP had been extensively utilized in alternative medicine because of its pharmacologically active compounds discovered in the plant’s parts, leaves, flowers, roots, and its milky latex [25, 26]. CP had been investigated to contain some important compounds which includes trierpenoids, anthocyanins, norditerpenic esters, organic acid, cysteine protease procerain, alkaloids, phenol, flavonoids cardenolides [27, 28]. Hence, this review focused on the CD146+ expression T cells (or lymphocytes), apoptosis of T cells and lung cancer, binding partners of CD146+ , molecular signaling of CD146+, CD146+ a novel marker of lymphocytes subset population, immunophenotyping and detection of CD146+, CD146+ T cells in cancer and effects of methanol extract of *Calotropis procera* leaf on CD146 expression. Investigation unfolding the molecular mechanism and regulation of CD146+ expression on the T cells is still limited.

## Main text

### Apoptosis of T cells and lung cancer

Apoptosis is a biochemical, physiological and pathological process that is involved in the regulation of the homeostasis. It regulates cell number in tissues and also eradicates distinct cells that intimidate animal survival [29]. It is essential in the organism due to the fact that inadequate apoptosis may results in lung cancer. Apoptosis occurring from activation of T cells is believed to help as a feedback mechanism that removes activated T cells [30]. Dissimilar to immature thymocytes and renovated T cell lines, resting T cells are extremely resilient to apoptosis after early activation but become highly vulnerable [31–33]. Therefore, most investigations on activation induced cell death (AICD) have studied mainly the connections between the death receptors, CD95 (Fas) and tumor necrosis factor (TNF)-α receptor, with their agonists CD95L (Fas ligand) [29, 34, 35]. Inactive normal T cells express little or non-measurable levels of CD95 and CD95L, nevertheless mitogenic activation of primary T cells distinctly proliferates their expression [36, 37]. Extra participants of the TNF-α receptor family, such as TRAIL-R1 and TRAIL-R2, can also activate apoptosis in vulnerable cells after binding of their ligands [29]. Notwithstanding the significant function of the death receptors, developing suggestion shows that environmental components, such as nonlymphoid secreted factors and cytokines, can control apoptosis of activated T cells, thus highlighting the implication of the environment in the maintenance of T-cell homeostasis [38, 39]. Any disparity in the apoptotic procedure may lead to some possible diseases state situations, lymphocyte accumulation, lymphocyte depletion, and lung cancer.

**Fig. 1:**
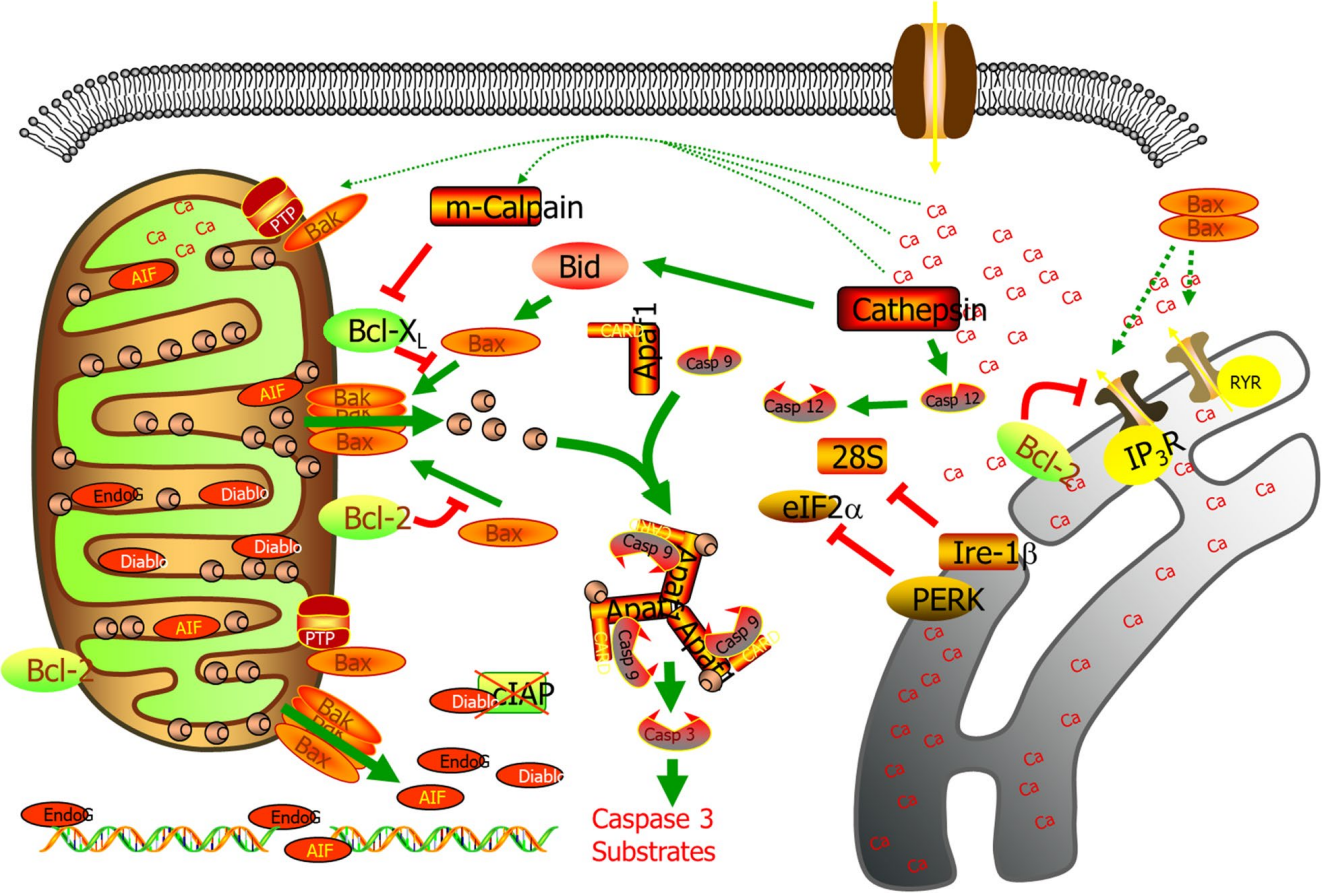
Mitochondria and ER Apoptosis. Variations in Ca^2+^ regulation and increase of misfolded proteins in the ER lead to ER stress that eventually results in apoptosis

Other causes involved in various forms of apoptosis are reactive oxygen species (ROS). Investigators have studied the connection of ROS in apoptosis of T-cell blasts and hybridomas by utilizing antioxidants such as *N*-acetyl cysteine and glutathione [40, 41]. Apoptosis in these cells is as a result of changes in mitochondrial permeability and following the release of ROS [42]. Investigations conducted on primary T cells, however, show that the formation of intracellular ROS is essential for T-cell activation and IL-2 secretion but also regulates activation-induced T-cell apoptosis, therefore proposing that intracellular ROS may possibly be involved in peripheral T-cell homeostasis [43–45]. Though, studies with primary T cells are frequently accomplished in cultures deficient of other “nonlymphoid” cells, although activation- induced T-cell apoptosis is assumed to happen in organs and tissues where other cell types, such as red blood cells (RBCs), are present [46]. Furthermore, the principal role is oxygen and CO_2_ transport [47]. Endoplasmic reticulum (ER) is responsible for intracellular calcium (Ca^2+)^ levels, protein folding, cellular responses to stress, protein synthesis, and trafficking [48]. Variations in Ca^2+^ regulation and increase of misfolded proteins in the ER lead to ER stress that eventually results in apoptosis (Fig. 1). Actually, the main factors that are responsible for apoptosis are the death receptor and the mitochondrial pathway [49]. The mechanism of ER stress-mediated apoptosis is assumed to utilize mitochondria, protein kinases, pro, and anti-apoptotic proteins heme oxygenase, microtubules, Ca^2+^, and caspases [48, 50–52]. ER stress-induced apoptosis which has been characterized include brefeldin, tunicamycin and thapsigargin [49]. Nevertheless, the investigation on the main mechanism of ER stress-induced apoptosis is still limited.

**Fig. 2:**
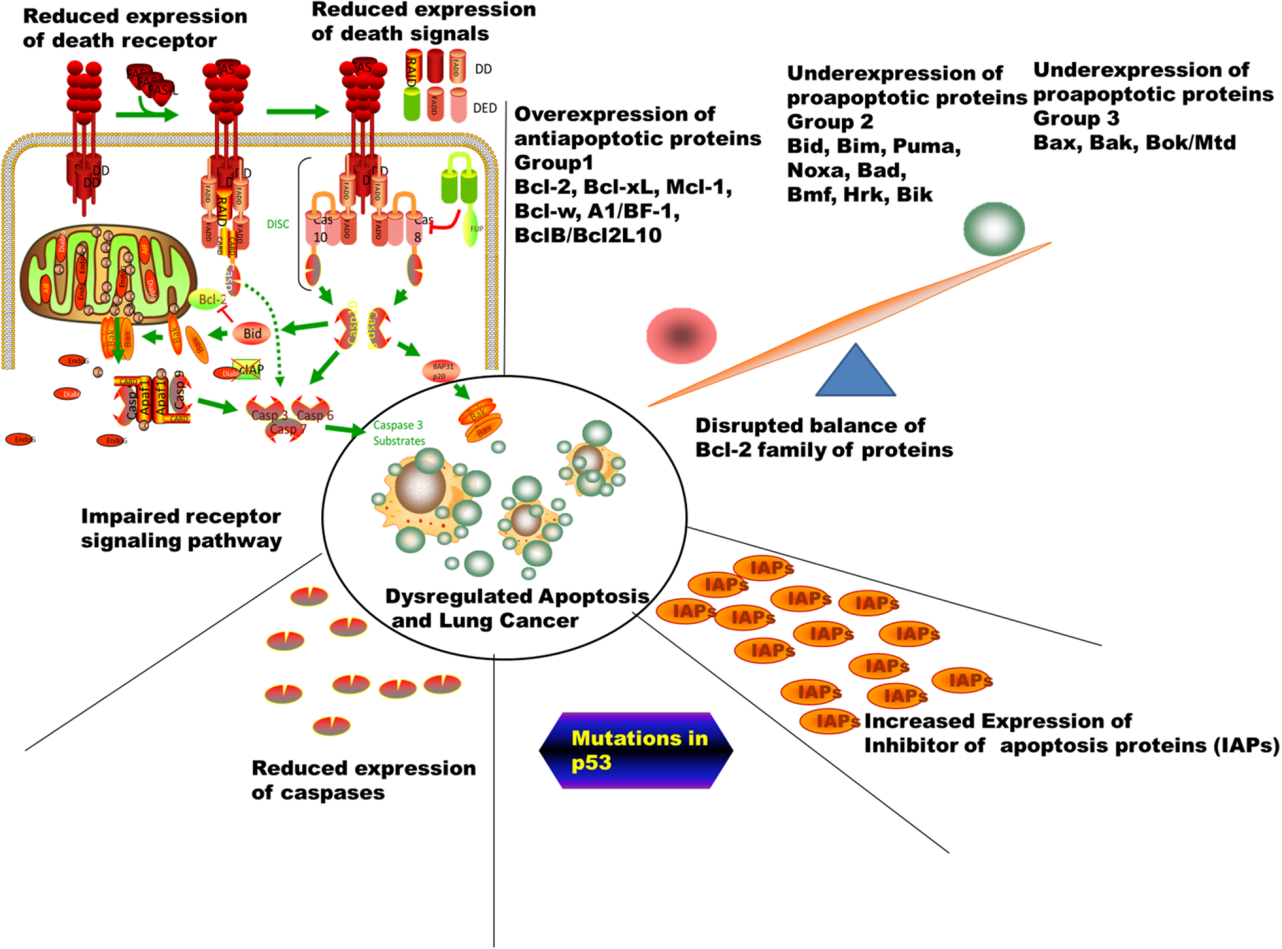
Dysregulated apoptosis and lung cancer. The anti-apoptotic and pro-apoptotic members of the Bcl-2 family undergo imbalance, which leads to dysregulated apoptosis. The overexpression of anti- apoptotic include group 1 (Bcl-2, Bcl-xL, Mcl-1, Bcl-w, A1/BF-1, BclB/Bcl2L10) and under expression of group 2 (Bim, Bid, Puma, Noxa, Bad, Hrk, Bik) and under expression of group 3 (Bak, Bax, and Bok/Mtd) resulted to dysregulated apoptosis and lung cancer. Other factors involved includes reduced expression of caspases, increased expression of inhibitor of apoptosis proteins, mutation on p53, impaired receptor signalling pathway and reduced expression of death receptor

Lung cancer can be regarded as a progression of genetic deviations during which a normal cell is changed into a malignant one whereas avoidance of cell death or apoptosis is one of the important factors in the lung cell that leads to malignant carcinogenesis [53]. Therefore, when there is insufficient or decreased apoptosis, it may lead to carcinogenesis. Lung malignant cell may procure a decrease in apoptosis. There are some major ways in which decrease in apoptosis may occur which includes the imbalance of the anti-apoptotic and pro-apoptotic proteins, decrease in caspase expression and malfunction of the death domain and reduced death receptor signaling. Investigations have been done on different proteins which indicate the presence of pro- or anti-apoptotic effects in the cell. Overexpression and under-expression have vital effects on lung cancer by decreasing apoptosis in the cancer cells. The Bcl-2 (B- cell lymphoma 2) proteins utilized the intrinsic pathway for the control of apoptosis and act in the upstream of molecular damage and take effect in the mitochondria [54]. There are different groups of Bcl 2, which includes group 1 (Bcl-B/Bcl2L10, A1/Bfl-1, Bcl-w, Bcl-2, Bcl-xL, and Mcl-1) which are anti-apoptotic proteins, group 2 (Bik Bid, Noxa, Bim, Bmf, Puma, Bad, Bmf, and Hrk) which are pro-apoptotic and responsible for endoplasmic reticulum stress, DNA damage, growth factor deficiency, group 3 (Bak, Bax, and Bok/Mtd) which are also pro-apoptotic [55]. The anti-apoptotic and pro-apoptotic members of the Bcl-2 family undergo imbalance, which leads to dysregulated apoptosis (Fig. 2). Human lung cancers are associated with a mutation in the p53 gene [56–58]. The Inhibitor apoptosis proteins (IAPs) include X-linked IAP (XIAP, BIRC4), BIRC8, BIRC7, apollon (BRUCE, BIRC6), surviving (BIRC5), c-IAP2 (BIRC3), c-IAP1 (BIRC2) and NAIP (BIRC1). They are characterized by utilizing the baculovirus IAPs domains. They are very useful during apoptosis and signal transduction. IAPs can prevent the caspase from binding to their substrates [59]. Caspases are very vital in the initiation (caspase-2, -8, -9 and -10) and execution (caspase-3, -6 and -7) of apoptosis. Hence, low levels of caspases expression or deficiency in caspase roles may result to reduce in apoptosis and lung carcinogenesis. Currently, there are a lot of drugs and small molecules which are manufactured based on the mechanisms of apoptosis which includes sodium butyrate, oblimersen sodium, depsipetide, HA14-1, fenretinide, ABT-263, flavopiridol gossypol, ABT-737, GX15-070, which direct action on the Bcl 2 proteins [60–64]. Some drugs and small molecules also target p53 and a lot of clinical trials are ongoing for some new drugs. A search at http://www.clinicaltrials.gov will reveal molecules and drugs that target numerous proteins associated with apoptosis such as Bcl 2 family, IAPS and p53.

### CD146 expression on the CD4+ Treg

**Fig. 3:**
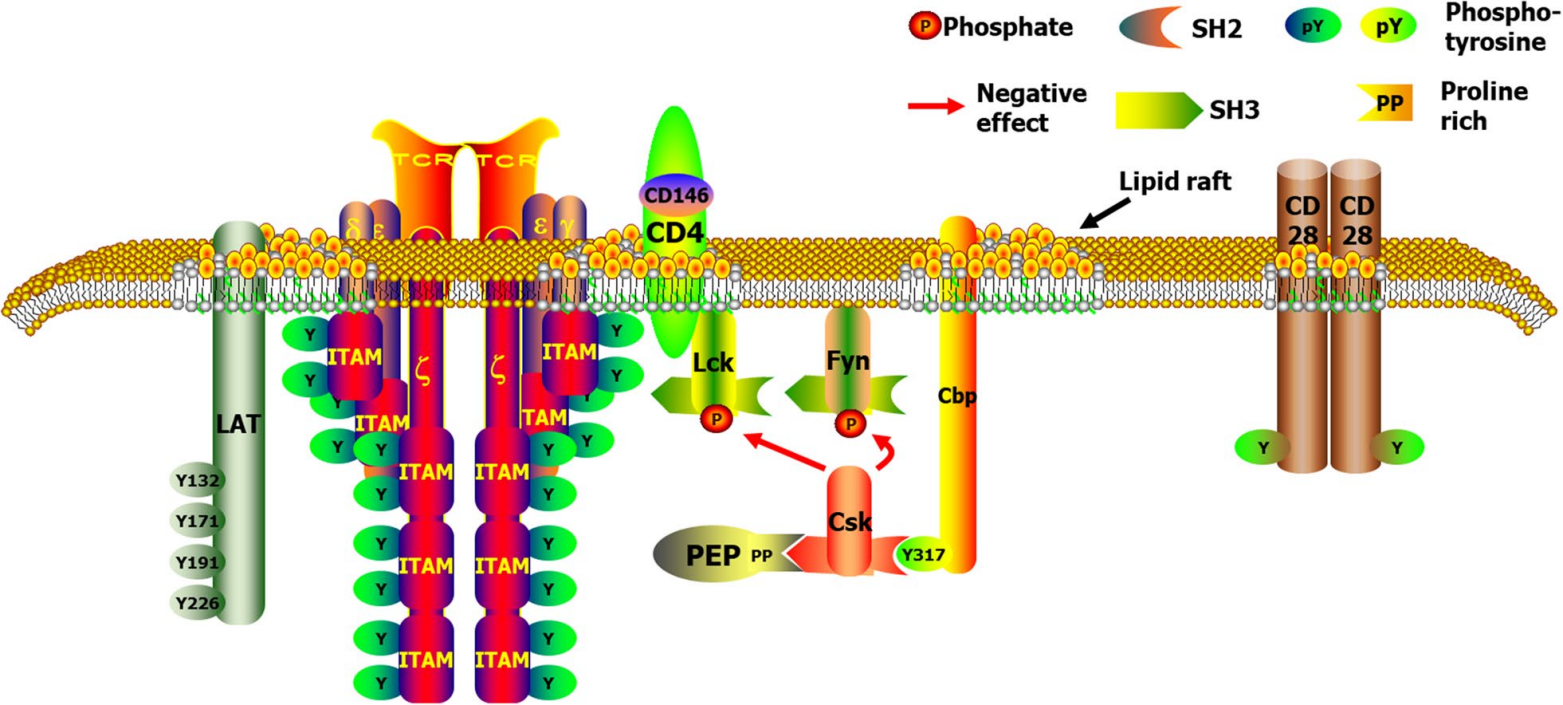
T Cell Receptor Complex and associated components. Showing the possible involvement of CD146 in cell signaling

CD146 formerly known as a melanoma marker [65], is an important glycoprotein found on the integral membrane of the cells. It is linked to the immunoglobulin superfamily with a feature V–V–C2–C2–C2 domain structure, and its cytoplasmic tail contains potential protein kinase C recognition sites and PDZ binding sites [66] indicating possible involvements in cell signaling (Fig. 3). CD146 mediates transduction of outside-in signals [67]. However, the precise extracellular ligand for CD146 is unclear. Sendo1 crosslinking with CD146, activate the phosphorylation of FAK through the link with Fyn [68]. Moreover, previous investigations discovered that CD146 mediates tumor secretion-induced p38/IkB kinase/nuclear factor-kB signaling cascade, which is essential in inducing endothelial cell activation, resulting in tumor angiogenesis [69–71]. Intracellular effectors and binding partners are very crucial in completely understanding of CD146 signaling. Treg cells may possibly also inhibit the antitumor immune responses. Predominantly in the environment of cancer, Treg-cell occurrences and roles are significant since increased numbers could results to tumor progression [72]. In most cancer patients, the immunophenotyping of Treg cells have concentrated primarily on co-expression of CD4+ and CD25+, while different types of Treg cell subtypes (Table 1) exist. To better comprehend and exploit Treg-cell biology in association with carcinogenesis and tumorigenesis, it will be very interesting to investigate more specific cell-surface markers.

**Table 1:** Characteristics of Subsets of T cells (Treg)

Subset	Specific marker	Secretory products	Actions	Location
nTreg	CD4, CD25, Foxp3	IL-10, TGF-β	Block T cell proliferation, suppression of DCs, inhibition of effector Th1, Th2, and Th17 cells; suppress mast cells, basophils, and eosinophils; interact with resident tissue cells	Thymus
nTeg	CD4, CD25, CD127	IL-10, TGF-β	Block T cell proliferation,	Neonatal thymus
ICOS(+) Treg	CD4, CD25, Foxp3, ICOS	IL-10, IL-17, IFN-γ	Suppress hapten-reactive CD8(+) T cells	Generated from nTregs
iTreg	CD4, Foxp3	IL-10, TGF-β	Similar to nTreg	Periphery
Tr1	CD4, CD25	IL-10	Suppress effector Th cell migration and functions suppress mast cells, basophils, and eosinophils	Generated from non-Treg cell precursors and home lungs and draining lymph nodes
CD8(+)Treg	CD8, Foxp3, CD25 (not for tonsil origin), CD28	IL-10, TNF-α, IFN-γ, GB	Block activation of naive or effector T cells; suppress IgG/IgE antibody responses [9], IL-4 expression and the proliferation of CD4(+) T cells	Generated from OT-1 CD8 cells [9] and tonsils
IL-17-producing Foxp3 (+) Treg	CD4, Foxp3, CCR6, RORGTF	IL-17	Inhibit the proliferation of CD4(+) effector T cells	Differentiated from CD4(+)Foxp3(+)CCR6(−) Tregs in peripheral blood and lymphoid tissue

*nTreg* natural regulatory T cell, *ICOS* inducible costimulator, *iTreg* inducible/adaptive regulatory T cell, *Tr1 cell* IL-10-producing type 1 regulatory T cell, *GB* granzyme B, *RORGTF* ROR gamma transcription factor

### Binding partners of CD 146

Previously CD146 was assumed to have a homotypic ligand-receptor interaction, a mechanism which is still unclear [20]. Recent investigations propose a ligand for CD146 is laminin-411 (α4-chain, a β1-chain, and a γ1 chain, also known as laminin-8) [73, 74]. Further investigations were conducted to show that CD146+ cells [75, 76] were self-regulated of very late antigen-4 (VLA-4) and in combination with p-selectin glycoprotein ligand-1 (PSGL-1)-mediated progressing of these cells. Galectin-1 (LGAL-1) and galectin-3 (LGAL3) have been shown to bind to CD146 [77]. Vascular endothelial growth factor receptor-2 (VEGFR2) and CD146 act together unswervingly and that this binding improves VEGFR2 signaling [78]. In endothelial cell and T cells, CD146 is a useful biomarker. It has importance in angiogenesis; nevertheless the molecular mechanism underlying angiogenesis remains unclear. CD146 and Wnt5a are binding partner [79]. The accounts of multiple ligands for CD146 are still debatable due to the mechanisms of adherence and migration of estimate the proportion of immune and cancer cells (EPIC) T cells. However, the investigations unfolding these ligands for CD146 have not been fully explored confirmed by subsequent studies and it is unclear if these ligands are competitive or cooperative. Further researches and explorations are required to explain the binding ligands of CD146 and the precise mechanism of migration and signaling in EPIC T cells.

### Molecular signaling of CD146

Molecular signaling of CD146 commitments have been extensively considered in numerous non-leukocytic cells types; but an inclusive, clear depiction of CD146 is interesting for investigation. Protein tyrosine kinase (PTK)-dependent signaling pathway, with tyrosine phosphorylation of the focal adhesion kinase, p125FAK, paxillin and NF-kB are important molecular signaling of CD146 [68, 80]. Investigations have been conducted on the reciprocal control of CD146 and Akt (serine/threonine specific protein kinase B) in melanoma cell lines. Akt is linked with tumor cell survival, proliferation, and invasiveness. The triggering of Akt is a variation detected in human cancer and tumor cells. Tumor cells that have regularly active Akt may be contingent on Akt for existence [81]. Therefore, Akt pathways may be possible therapies for cancer and tumor cells resulting in inactivation of the Bcl-2-associated death promoter (BAD).

Pervious Investigations [19] suggest utilizing human umbilical cord endothelial cells, as well as zebrafish embryos showed that CD146 binds to Wnt5a with high affinity and is important for endothelial cell migration and activity of c-jun amino-terminal kinase (JNK) via non-canonical signaling. The phosphorylation of Disheveled (Dvl), insulin-like growth factor binding protein 4 (IGFBP4), an opponent of the Wnt/β-catenin signaling, was discovered to trigger Wnt/β-catenin signaling pathway and to encourage the expression of CD146 in renal carcinoma cells. Currently, research is limited in the investigation of human T cells describing the signaling pathways associated with CD146 engagement.

### CD146 a novel marker of lymphocyte subset population

Initially, the expression of CD146 on lymphocyte appeared in 1997 [82]. Hence, the expression of CD146 on the leukocytes of healthy donors was not significant. It may be found in CD4+ and CD8+ subpopulation using TCRVβ analysis. The low percentage may be detected on B cells and sometimes in NK population. Moreover, skin specimens from contact dermatitis patients established that 50–80% of the CD3+ cells in tissue sections were CD146+. This initial investigation lay quiescent for nearly a decade until another researcher identified CD146+ T cells in the peripheral blood circulation of healthy donors [6]. CD146 could be upregulated on B cells by mitogen stimulation such as PMA, and by activation with a combination of CD40L and IL-4 [6]. The utilization of the techniques of immunohistochemistry has demonstrated the presence of CD146 on immature cortical thymocytes, confirming the idea that this antigen was expressed on T cells at an early stage [83]. It was also discovered in the peripheral blood of Treg cells in the lung cancer patient which was significantly different from control (healthy subjects).

### Immunophenotyping and detection of CD146

**Fig. 4:**
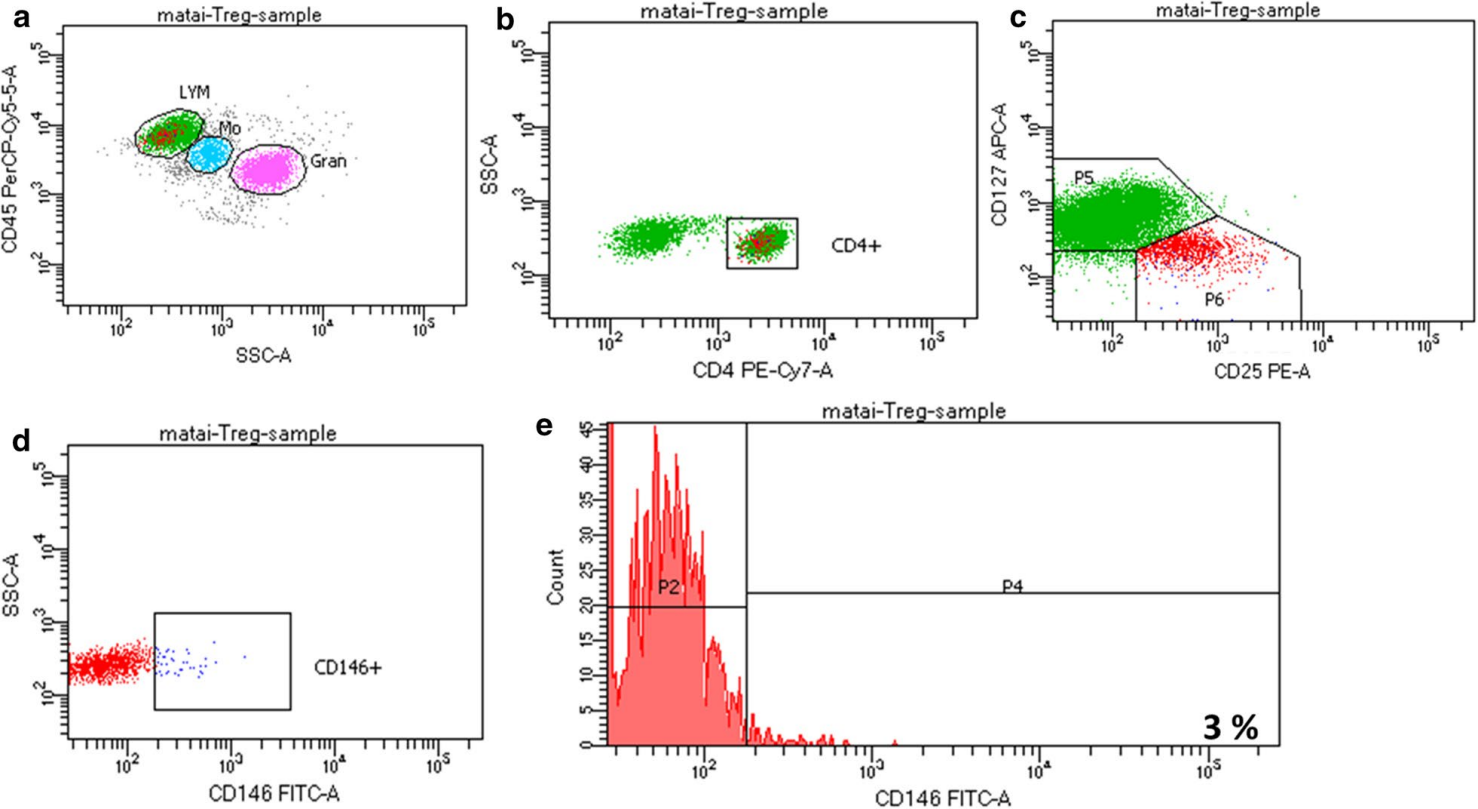
Expression of CD 146+ in healthy subject. **a** CD45PreCP-cy5-5-A vs SSCA. **b** SSC-A vs CD4PE-Cy7-A. **c** CD127 APC-A vs CD 25 PE-A. **d** SSCA vs CD 146 FITC-A. (E) Count vs CD 146 FITC-A = 3%

**Fig. 5:**
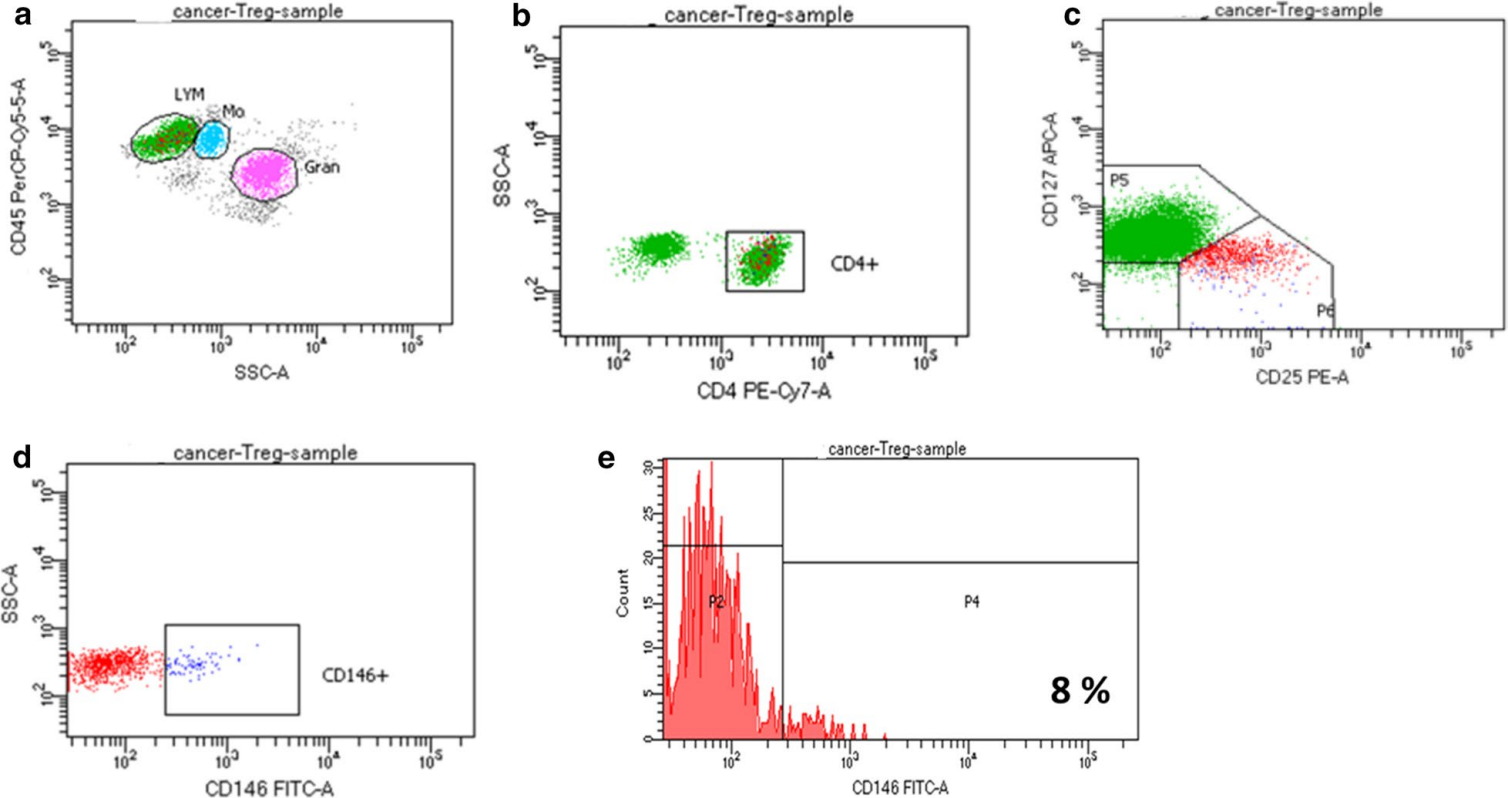
Expression of CD 146+ in lung cancer patient. **a** CD45PreCP-cy5-5-A vs SSCA. **b** SSC-A vs CD4PE-Cy7-A. **c** CD127 APC-A vs CD 25 PE-A. **d** SSCA vs CD 146 FITC-A. **e** Count vs CD 146 FITC-A = 8%

Immunophenotyping of CD146 is the exploration of heterogeneous populations of CD146 in the T cells for identifying the presence, expression, and proportions. Nevertheless, CD146 was previously designated as an activation antigen of T cells, the circulation of this antigen is different from other common markers of activation such as, OX-40, CD38, CD25, HLA-Dr and CD69 in freshly isolated cells [19]. However, there are different amount of the expression of CD146 with many of the other activation markers [84]. CD146+ T cells were also discovered to be CCR7−, CD28+, CD45RA−, CD45RO+, designated as effector memory T cells [66]. Markers linked with Th17 cells, CD58 and CD26 were concurrently expressed on CD146 positive cells, but extra markers associated with Th17 cells, including, CCR4 and CCR6 were moderately expressed with CD146 [76]. Markers associated with Treg cells, CD4+, CD25+ and CD127dim/- were simultaneously expressed on the CD146 positive cells in healthy subject Fig. 4 and lung cancer patient Fig. 5 respectively.

**Fig. 6:**
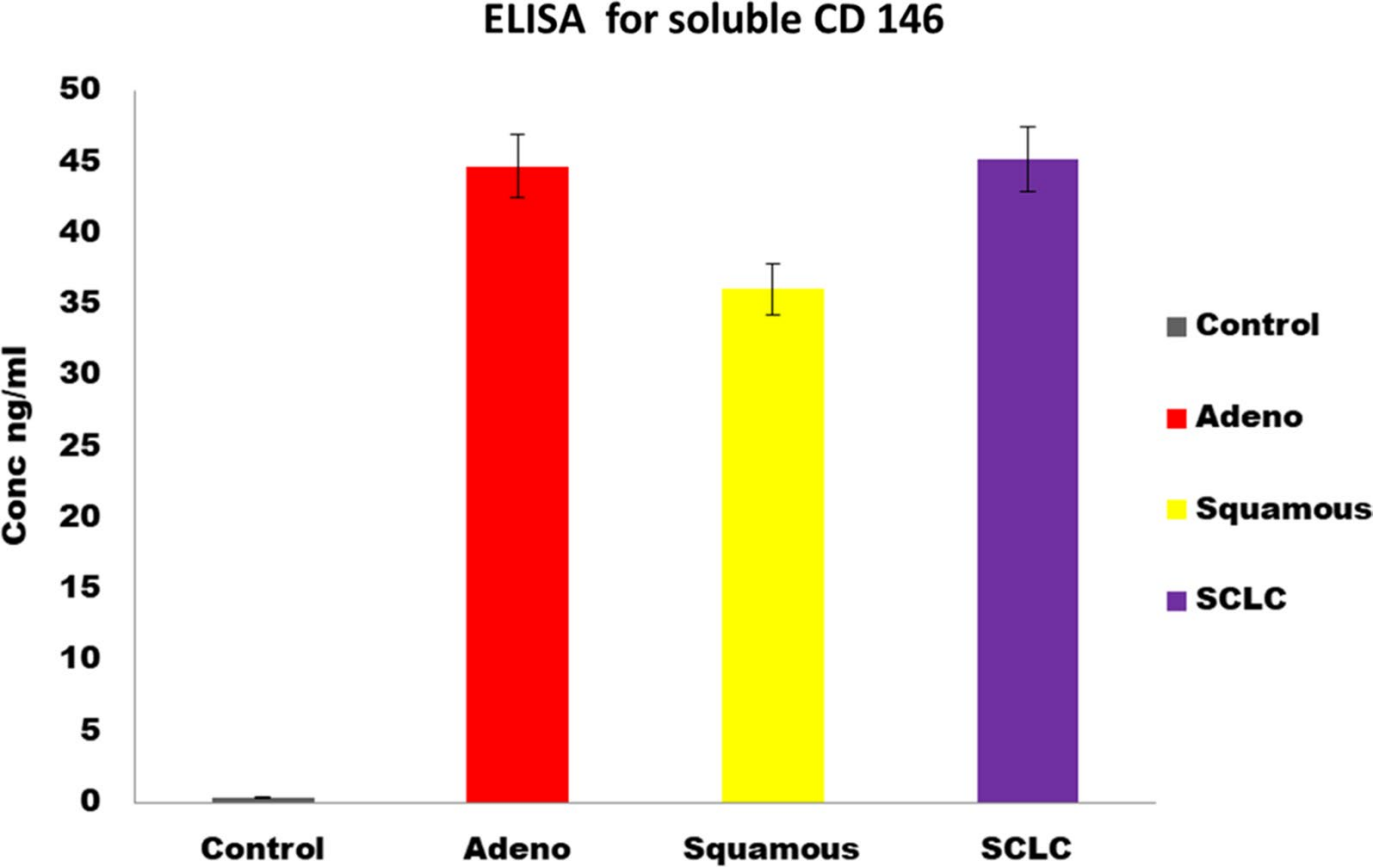
Detection of sCD146 using ELISA. The expression of sCD 146 was significantly higher in patients than in controls (P < 0.05). From the ELISA, the concentration of the sCD146 includes 0.33 ± 0.09 ng/ml in healthy patients (Control), 44.69 ± 0.29 ng/ml, 36.05 ± 0.24 ng/ml, 45.18 ± 0.27 ng/ml in adenocarcinoma, squamous and small cell lung cancer, respectively

CD146 can be detected utilizing the following techniques. These include flow cytometry, immunofluorescence, immunohistochemically staining of paraffin-embedded tumor samples, electron microscopy, confocal microscopy, application of a chemiluminescence detection system [82], immunomagnetic sorting, immunoprecipitation, mass spectrometry, western blot [82, 85], ELISA can be used for the detection of soluble CD146 from serum or plasma Fig. 6 [11], real-time PCR of CD146 mRNA [86] and quantitative real-time PCR can also be utilized for the detection of CD146 [87, 88].

### CD146 T cells in cancer

CD146 is a multifunctional molecule that contributes in various molecular, biochemical, physiological [89] and pathological processes relating to immunity, signal transduction, stem cell differentiation [75, 90] and angiogenesis [3, 91]. In recent years, various investigations revealed that CD146 overexpression significantly relates with the metastasis, progression and formation of new blood vessels of some malignant tumors which was investigated in melanoma, esophageal cancer, prostate cancer, gallbladder adenocarcinoma, ovarian carcinoma [87, 88, 92–101]. Currently, researchers are conducting investigations on the different kinds of cancer and the output revealed the different correlations of CD146 with cancer. Hence, high number of investigation indicated that CD146 is highly expressed in solid tumors, including lung cancer [12, 13, 102–104], hepatocellular carcinoma [105, 106], epithelial ovarian cancer [107], breast cancer [108, 109], leiomyosarcoma [110], esophageal squamous cell carcinoma [111], gallbladder adenocarcinoma [98], colorectal cancer [112], gastric cancer [113], clear cell renal cell carcinoma [114], melanoma [115], hematological malignancies [116], peripheral nerve tumors [117], parotid carcinoma [118], non-small cell lung cancer [12], infantile haemangioma [119], adenoid cystic carcinoma [120], malignant pleural mesothelioma [121], pancreatic cancer [14], prostate cancer [122], cervical cancer and endometrium cancer [123]. The different kinds of cancer and the implications of CD146 have been summarized in Table 2. These obvious suggestions on the expression of CD146 in cancer indicated that the transmembrane glycoprotein would be further deliberated as a potential biomarker for the diagnosis of cancer patients and therapeutic target.

**Table 2:** The implication of increased CD146 in Cancer

Cancer type	Implications	References
Lung cancer	Poor prognosis	[12, 13, 102–104]
Hepatocellular carcinoma	Promotes metastasis and predicts poor prognosis	[105, 106]
Epithelial ovarian cancer	Poor prognosis	[107]
Breast cancer	Elevated epithelial-mesenchymal transition	[108, 109]
Leiomyosarcoma	Prognostic factor	[110]
Esophageal squamous cell carcinoma	Poor prognosis	[111]
Gallbladder adenocarcinoma	Progression, metastasis, and poor-prognosis	[98]
Colorectal cancer	Poor prognosis	[112]
Gastric cancer	Poor prognosis	[113]
Clear cell renal cell carcinoma	Elevated reoccurrence	[114]
Melanoma	Elevated metastasis and poor prognosis	[115]
Hematological malignancies	Elevated tumorigenesis	[116]
Peripheral nerve tumors	Modulator of malignant transformation	[117]
Parotid carcinoma	Elevated progression and invasion	[118]
Non-small cell lung cancer	Poor prognosis	[12]
Infantile haemangioma	Elevated progression	[119]
Adenoid cystic carcinoma	Elevated progression	[120]
Malignant pleural mesothelioma	Poor prognosis	[121]
Pancreatic cancer	Poor Prognosis and cancer progression	[14]
Cervical cancer	Dissemination and metastasis	[123]
Endometrium	Dissemination and metastasis	[123]
Prostate cancer	Poor prognosis	[122]

### Effects of methanol extract of *Calotropis procera* leaf on CD146 expression

**Fig. 7:**
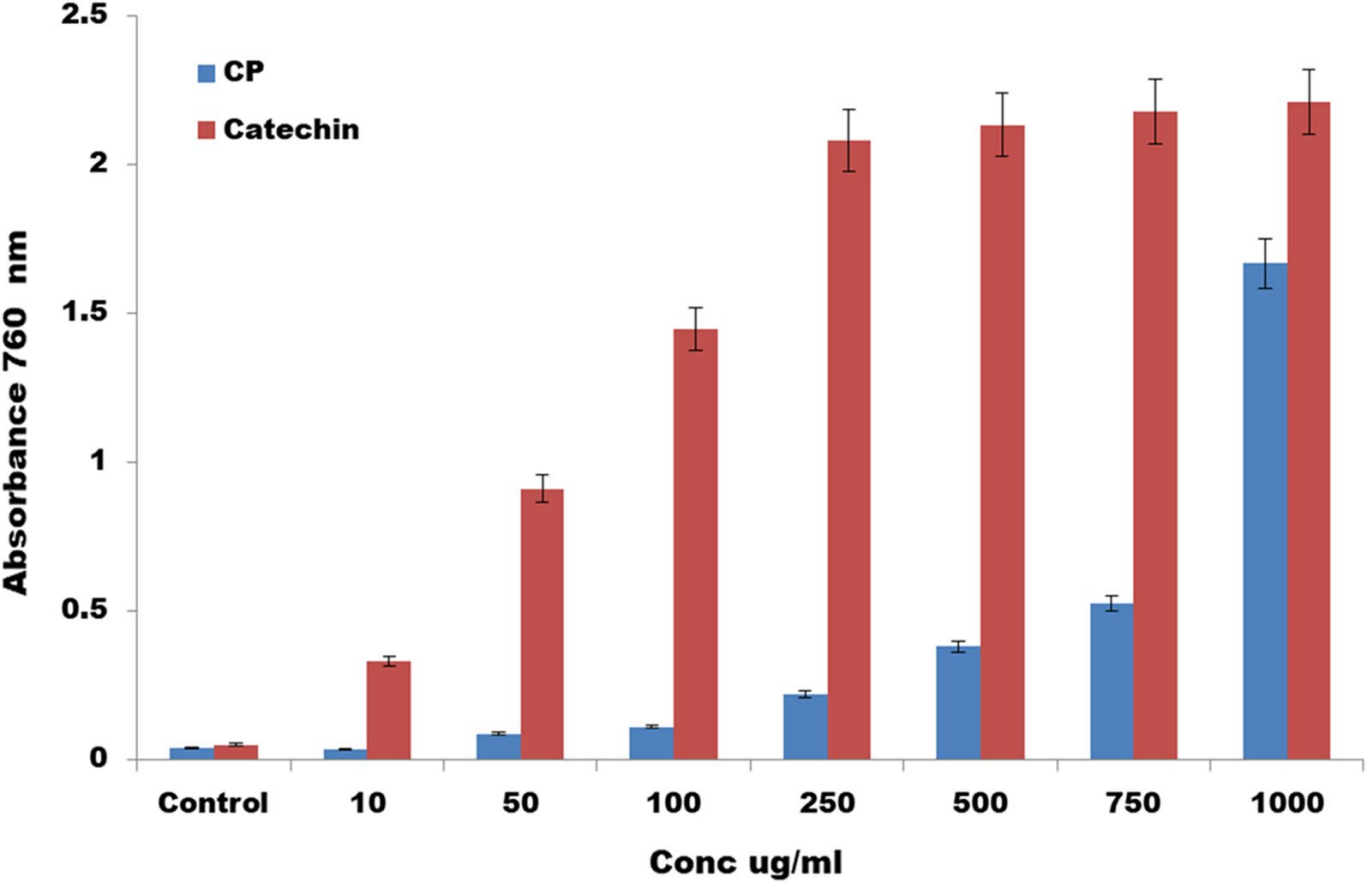
Total Phenolic content for *Calotropis Procera leaf*. It revealed that at 1000 ug/ml, the phenolic content was similar to the standard catechin

**Fig. 8:**
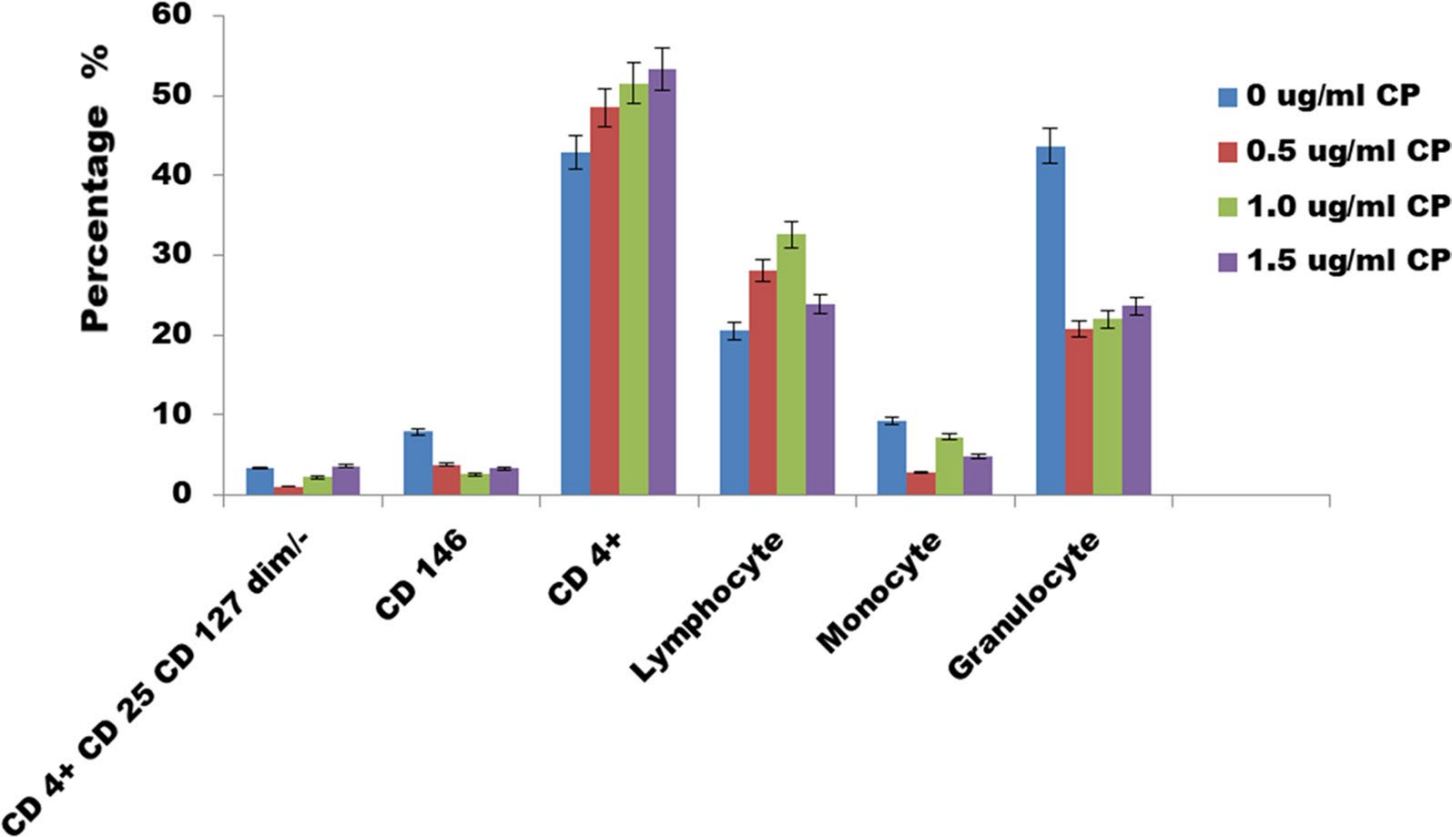
Effects of CP on CD 146 at 12 hours. CP was used on the blood cells and we discovered that it reduced the expression of CD146, increased the expression of CD4+ and dose dependent. It shows that CP may be an immunotherapy

In this review, an effect of methanol extract of *Calotropis procera* leaf on CD146 expression was explored to discover that it has phenolic contents Fig. 7. It revealed that at 1000 ug/ml, the phenolic content in CP was similar to the standard catechin which indicates that it is an antioxidant. CP was used on the blood cells and we discovered that it reduced the expression of CD146 Fig. 8; hence it may be a potential immunotherapy for the treatment of cancer and various diseases. The CD4+ cells increased and it was dose dependent. Hence it is very interesting to unravel the dose of CP which may be used for the treatment of cancer induced animal models. Further investigations are also required to find out the molecular mechanism that is responsible for the reduction of CD146 using the CP.

## Conclusion

Currently, there is no data to describe the genetic association of CD146 expression and IL-17 secretion. However, some IL-17 secreting T cells can be discovered in healthy individuals without CD146 expression on those cells. T cells may be potential immunotherapy for lung cancer and other types of cancer. Targeting of the T cells and CD146 when the ligand is known through the migration of these cells to sites of injury or tumor cells is interesting. However, investigation unfolding the molecular mechanism and regulation of CD146 expression on the T cells is still limited and is a hot topic for investigations. CD146, apoptosis of the T cells and lung cancer are also of very great significance because these may be excellent target for the treatment of carcinogenesis. Future investigations to unravel the significance of methanol extract of *Calotropis procera* leaf on CD146 expression will be very interesting. Utilization of cancer cell lines, animal models and using CP as a therapy will be fascinating. Hence the molecular mechanism underlying the process by which CP ameliorate the expression of CD146 will be of unique importance to the investigation. Therefore CD146 is a molecule of significance which can also be studied in other diseases state such as inflammation, COPD, pulmonary arterial hypertension and other respiratory diseases. Animal models of PAH and other diseases model or knockout mouse can be investigated to unravel the expression of CD146 in these models and comparisons with the human samples can also be conducted to unveil the possible diagnosis, therapy and prognosis.

## Data Availability

Not applicable.

## Abbreviations

VEGF: vascular endothelial growth factor
IL-10: interleukin 10
IL-4: interleukin 4
IL-17: interleukin 17
NF-κB: nuclear factor-kappa B
IL-1R: interleukin-1 receptor
(TNF)-α receptor: tumor necrosis factor -α receptor
Akt: serine/threonine specific protein kinase B
CD: cluster of differentiation
LGAL 1: galectin 1
LGAL 3: galectin 3
